# MRI Radiomics for Predicting the Diffuse Type of Tenosynovial Giant Cell Tumor: An Exploratory Study

**DOI:** 10.3390/diagnostics15182399

**Published:** 2025-09-20

**Authors:** Seul Ki Lee, Min Wook Joo, Jee-Young Kim, Mingeon Kim

**Affiliations:** 1 Department of Radiology, St. Vincent’s Hospital, College of Medicine, The Catholic University of Korea, Seoul 06591, Republic of Korea; 2 Department of Orthopaedic Surgery, St. Vincent’s Hospital, College of Medicine, The Catholic University of Korea, Seoul 06591, Republic of Korea; 3 Research Collaboration, Siemens Healthineers Ltd., Seoul 06620, Republic of Korea

**Keywords:** tenosynovial giant cell tumor, radiomics, diffuse-type, magnetic resonance imaging, machine learning

## Abstract

**Objective:** To develop and validate a radiomics-based MRI model for prediction of diffuse-type tenosynovial giant cell tumor (D-TGCT), which has higher postoperative recurrence and more aggressive behavior than localized-type (L-TGCT). The study was conducted under the hypothesis that MRI-based radiomics models can predict D-TGCT with diagnostic performance significantly greater than chance level, as measured by the area under the receiver operating characteristic (ROC) curve (AUC) (null hypothesis: AUC ≤ 0.5; alternative hypothesis: AUC > 0.5). **Materials and Methods:** This retrospective study included 84 patients with histologically confirmed TGCT (54 L-TGCT, 30 D-TGCT) who underwent preoperative MRI between January 2005 and December 2024. Tumor segmentation was manually performed on T2-weighted (T2WI) and contrast-enhanced T1-weighted images. After standardized preprocessing, 1691 radiomic features were extracted, and feature selection was performed using minimum redundancy maximum relevance. Multivariate logistic regression (MLR) and random forest (RF) classifiers were developed using a training cohort (*n* = 52) and tested in an independent test cohort (*n* = 32). Model performance was assessed AUC, sensitivity, specificity, and accuracy. **Results:** In the training set, D-TGCT prevalence was 32.6%; in the test set, it was 40.6%. The MLR model used three T2WI features: wavelet-LHL_glszm_GrayLevelNonUniformity, wavelet-HLL_gldm_LowGrayLevelEmphasis, and square_firstorder_Median. Training performance was high (AUC 0.94; sensitivity 75.0%; specificity 90.9%; accuracy 85.7%) but dropped in testing (AUC 0.60; sensitivity 62.5%; specificity 60.6%; accuracy 61.2%). The RF classifier demonstrated more stable performance [(training) AUC 0.85; sensitivity 43.8%; specificity 87.9%; accuracy 73.5% and (test) AUC 0.73; sensitivity 56.2%; specificity 72.7%; accuracy 67.3%]. **Conclusions:** Radiomics-based MRI models may help predict D-TGCT. While the MLR model overfitted, the RF classifier demonstrated relatively greater robustness and generalizability, suggesting that it may support clinical decision-making for D-TGCT in the future.

## 1. Introduction

Tenosynovial giant cell tumor (TGCT) is a rare, benign, but locally aggressive neoplasm that arises from the synovial lining of joints, bursae, and tendon sheaths [1]. With an estimated annual incidence of 1.8 to 50 cases per million, TGCT primarily affects young to middle-aged adults, often causing significant morbidity. Although histologically benign, TGCT exhibits variable clinical behavior, ranging from indolent nodules to extensively infiltrative masses. According to the World Health Organization (WHO) classification of soft tissue and bone tumors, TGCT is categorized into two distinct subtypes: localized-type (L-TGCT) and diffuse-type (D-TGCT) [2]. L-TGCT is far more common and typically presents as a well-circumscribed nodule with indolent behavior, often amenable to local excision with favorable outcomes. In contrast, D-TGCT represents a minority of cases and is characterized by diffuse synovial proliferation with poorly defined margins, frequently involves large joints such as the knee, and is associated with higher rates of postoperative recurrence, joint destruction, and significant functional impairment. The high propensity for local recurrence after surgical excision further complicates management, with some reported recurrence rates as high as 40–60% [3]. These characteristics can necessitate multimodal management— such as extensive surgical interventions and, in some cases, adjunctive or systemic therapies—thereby increasing treatment complexity and healthcare burden [1,4,5]. Consequently, accurate identification of D-TGCT is critical for optimal treatment planning and prognostic assessment [1,6].

Magnetic resonance imaging (MRI) is the preferred imaging modality for evaluating TGCT due to its superior soft tissue contrast and multiplanar capability. MRI plays a key role in assessing tumor extent, evaluating adjacent joint and bone involvement, and guiding treatment planning [1]. Previous studies have identified certain imaging findings that may help distinguish between L-TGCT and D-TGCT; for example, multinodular lesions with poorly defined, infiltrative margins reflect the aggressive synovial proliferation of D-TGCT [5]. The absence of a peripheral hypointense rim on T2-weighted imaging (T2WI), commonly seen in localized-type tumors, has been suggested as another discriminative feature for diffuse-type tumors [7]. D-TGCT often demonstrates granular internal hypointensity on T2WI due to hemosiderin deposition [7]. Involvement of adjacent bone, cartilage erosion, and extra-articular extension are more frequently observed in D-TGCT, indicating its invasive potential and correlating with poorer prognosis [4,5]. These features are also associated with disease severity and recurrence risk [5,6,7]. Some studies have explored advanced imaging sequences, such as diffusion-weighted imaging (DWI) and dynamic contrast-enhanced MRI (DCE-MRI), to improve diagnostic accuracy [8,9], but these techniques have not yet achieved widespread clinical adoption due to inconsistent results and technical limitations.

Despite these diagnostic advances, the differentiation between L-TGCT and D-TGCT based on conventional MRI remains challenging. The subjective interpretation of these qualitative imaging features, such as infiltrative margins, often leads to interobserver variability and diagnostic ambiguity, which can delay or compromise appropriate treatment decisions. Ultimately, definitive diagnosis requires histopathologic confirmation obtained after surgical excision, which is invasive and not available at the time of treatment planning. These shortcomings highlight the need for reliable, noninvasive imaging biomarkers capable of objectively predicting D-TGCT.

In this context, radiomics has emerged as a promising quantitative imaging approach that can overcome these limitations by providing a more objective and data-driven method. Radiomics enables the high-throughput extraction of a large number of quantitative features from medical images, capturing tumor heterogeneity, shape, intensity, and texture that may not be visually discernible [10]. Applied to conventional MRI, radiomics has demonstrated potential in tumor classification, prognosis prediction, and treatment assessment across various oncologic contexts [11,12]. For example, in non-small cell lung cancer, radiomics-based models have been used to predict tumor histology, genetic mutations such as EGFR (Epidermal Growth Factor Receptor) status, and patient prognosis [13]. In glioblastoma, radiomics signatures from multiparametric MRI correlated with molecular subtypes and predicted survival outcomes more accurately than conventional imaging alone [14]. In breast cancer, radiomics combined with machine learning algorithms improved discrimination between benign and malignant lesions on MRI, reducing unnecessary biopsies [15]. In prostate cancer, multiparametric MRI radiomics enhanced lesion detection, aggressiveness grading, and treatment response monitoring [16]. These advances illustrate how radiomics and machine learning can provide objective imaging biomarkers that support diagnosis, prognostication, and therapeutic decision-making. While a few studies have explored radiomics in musculoskeletal (MSK) tumors [17,18], its application in TGCT remains underexplored. To our knowledge, no prior study has systemically investigated whether MRI-based radiomics can distinguish D-TGCT from L-TGCT. Accurate distinction between these subtypes is clinically relevant, as it directly influences treatment approach and prognosis.

The purpose of this study was to develop and validate a radiomics-based predictive model using MRI to assess the risk of D-TGCT. We selected T2WI and contrast-enhanced T1-weighted imaging (CE T1WI) because these are routinely acquired, provide complementary tissue contrast, and capture distinct tumor characteristics—T2WI reflecting lesion heterogeneity and fluid content, and CE T1WI highlighting vascularized tumor components. By extracting and analyzing quantitative features from T2WI and CE T1WI, we aimed to create a robust radiomics-based predictive model capable of reliably discriminating D-TGCT. We anticipate that this approach will provide insights for the earlier risk stratification of patients with D-TGCT, supporting surgical planning and potentially guiding further research toward more personalized treatment strategies. The primary hypothesis of this study was that MRI-based radiomics models can predict D-TGCT with diagnostic performance significantly greater than random chance. The null hypothesis was that the radiomics models would not be able to effectively differentiate D-TGCT from L-TGCT. Conversely, the alternative hypothesis was that the radiomics models could successfully differentiate between D-TGCT and L-TGCT, potentially providing a supportive tool for future clinical decision-making, pending further validation.

## 2. Materials and Methods

This retrospective study was approved by the institutional review board of our institution, and the requirement for informed consent was waived due to its retrospective nature.

### 2.1. Study Population

**Figure 1:**
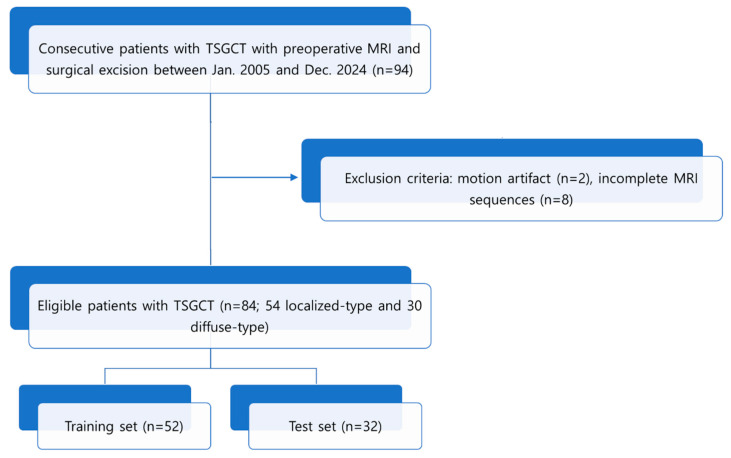
Flowchart of patient inclusion. TGCT, tenosynovial giant cell tumor.

This retrospective study included 94 patients with histologically confirmed TGCT who underwent preoperative MRI between January 2005 and December 2024 at a single tertiary referral center. They were included based on the following inclusion criteria: (1) preoperative MRI, (2) surgical excision, and (3) no prior treatment such as surgery, radiation, or systemic therapy. Ten cases were excluded due to the following exclusion criteria: two cases with severe motion artifacts that compromised image quality, and eight cases with incomplete MRI sequences. In total, 84 patients with histologically confirmed TGCT, comprising 54 cases of L-TGCT and 30 cases of D-TGCT, were finally enrolled. Demographic data, including sex and age, were obtained from the medical records. A flowchart of the patient selection process is shown in Figure 1.

### 2.2. MRI Acquisition

All MRI examinations were performed with 1.5-T (Ingenia, Philips Healthcare, Best, The Netherlands) or 3.0-T scanners (Magnetom Verio or Magnetom Vida, Siemens Healthineers, Erlangen, Germany). The imaging protocol included the following sequences: spin-echo T1-weighted (TR/TE range, 370–693/10–19 in 1.5-T, 623/11 in 3.0-T), spin-echo T2WI with and without fat suppression (TR/TE range: 1648–3280/80–100 in 1.5-T, 4000–6200/63–76 in 3.0-T), and CE T1WI with fat suppression following intravenous administration of a gadolinium-based contrast agent (0.1 mmol/kg). All images were acquired in axial, coronal, and sagittal planes. Slice thickness ranged from 3 to 5 mm, with interslice gaps of 0–0.5 mm. Standardized positioning and field-of-view parameters were maintained to ensure consistency across subjects.

### 2.3. Tumor Segmentation

Tumor segmentation was manually performed using ITK-SNAP software (version 3.8.0; (http://www.itksnap.org/ accessed on 1 August 2025) [19]. The segmentation process was carried out on both T2WI and CE T1WI sequences. A musculoskeletal radiologist with nine years of experience (S.K.L.) first delineated the tumor margins using a free-hand tool on each axial image slice, defining the volume of interest (VOI). During tumor segmentation, only tumor tissue was included within the VOI, while adjacent surrounding tissues such as synovium, edema, and reactive inflammation were carefully excluded to ensure the specificity of radiomics analysis. Segmentation was then reviewed and validated by a second musculoskeletal radiologist with 31 years of experience (J.Y.K.). Both readers were blinded to the tumor subtype (L-or D-TGCT) during segmentation to minimize bias. To assess interobserver agreement, the radiologist performed tumor segmentation again for each patient more than a month after completing initial segmentation.

### 2.4. Radiomics Feature Extraction

Radiomics analysis was performed using Syngo.Via Frontier software (Version 1.2.2; Siemens Healthineers, Erlangen, Germany). This software package was developed based on the PyRadiomics library, version 3.0.1 (https://github.com/Radiomics/pyradiomics accessed on 1 August 2025), and scikit-learn machine learning library (https://scikit-learn.org/stable/modules/generated/sklearn.ensemble.RandomForestClassifier.html accessed on 1 August 2025). Both T2WI and CE T1WI sequences, along with the corresponding segmentation masks, were loaded into the software.

Prior to feature extraction, all images underwent standardized preprocessing to ensure consistency across different MRI acquisitions. Images were resampled to an isotropic voxel size of 1 × 1 × 1 mm^3^ using B-spline interpolation. This interpolation method was selected for its superior ability to maintain image smoothness and spatial integrity compared to other interpolation techniques, thereby minimizing resampling-induced artifacts that could bias texture analysis [20]. Intensity normalization was applied using z-score transformation to mitigate intensity variation due to scanner differences or acquisition parameters, enabling improved comparability of radiomics features [21]. Subsequently, image intensities were discretized using a fixed bin width of 25, a parameter chosen to balance the preservation of meaningful texture patterns against noise suppression during histogram-based feature calculation.

A total of 1691 radiomics features were extracted per image sequence, spanning multiple categories. These included first-order statistics that quantify basic intensity distribution characteristics, such as mean, variance, skewness, and kurtosis; shape-based descriptors measuring geometric properties like sphericity, compactness, and surface area; and a variety of texture features derived from established matrices, including the Gray Level Co-occurrence Matrix (GLCM), Gray Level Run Length Matrix (GLRLM), Gray Level Size Zone Matrix (GLSZM), and Neighboring Gray Tone Difference Matrix (NGTDM) [10]. To capture multi-scale tumor heterogeneity, wavelet-filtered images were also processed, providing features that reflect both coarse and fine texture patterns.

### 2.5. Feature Selection and Radiomics Model Development

Given the high dimensionality of the radiomic dataset and the risk of overfitting, feature selection was conducted using the minimum redundancy maximum relevance (mRMR) algorithm based on the R^2^ difference. The mRMR method mathematically optimizes feature subsets by selecting features that maximize mutual information with the outcome variable (D-TGCT) while minimizing redundancy between features, thus ensuring a compact yet informative feature set that enhances model interpretability and predictive power [22].

Following feature selection, prediction models were developed using two machine learning algorithms: multivariate logistic regression (MLR) and random forest (RF). For the MLR model, the number of features was further limited by the “one-in-ten rule,” which recommends including no more than one predictor per 10 outcome events to maintain statistical stability and avoid overfitting in models with limited sample sizes [23]. This rule is well validated in clinical prediction research to improve model generalizability.

The RF model classifier was developed using the full feature set and optimized through a rigorous hyperparameter tuning process embedded in a tenfold internal cross-validation framework [24]. The training data were randomly split into 10 subsets, iteratively training the model on nine folds and validating on the remaining fold. This cross-validation cycle was repeated 10 times, ensuring that each subset served as validation once [25]. Hyperparameters, including the number of trees, maximum tree depth, and minimum samples per split, were systematically adjusted using grid search to identify the combination that achieved the best balance between bias and variance. This robust tuning procedure mitigates overfitting and improves model stability.

The dataset was divided into a training cohort (52 patients, 65%), with class distributions balanced to maintain representativeness, and an independent test cohort (32 patients, 35%) reserved for unbiased validation of model performance.

### 2.6. Model Validation

**Figure 2:**
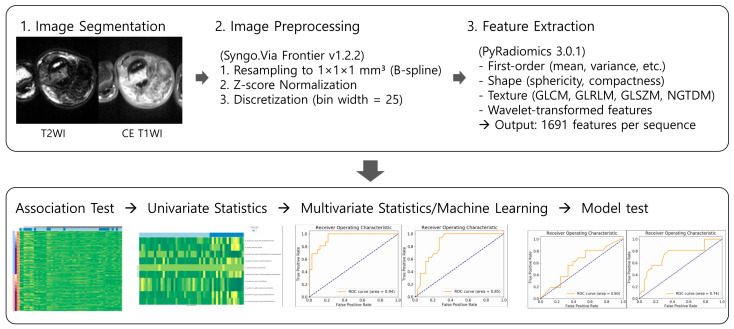
Radiomics pipeline of our study. The images included in the diagram were captured from the software we used: Syngo. Via Frontier Radiomics (Siemens Healthineers, Erlangen, Germany).

Model performance was assessed on the independent test cohort (*n* = 32) using the following evaluation metrics: area under the receiver operating characteristic (ROC) curve (AUC), sensitivity, specificity, and accuracy. The AUC is a widely used measure of the performance of supervised classification rules, which can be applied for multiclass classification problems [26]. The Youden index was used to identify optimal cutoff values from the training set, which were then applied to the test set. Comparative ROC curves for both MLR and RF models were generated to visually assess diagnostic performance differences. The entire radiomics workflow, from image preprocessing to model validation, is summarized in Figure 2.

### 2.7. Statistical Analysis

The primary hypothesis of this study was that MRI-based radiomics models can predict D-TGCT with diagnostic performance significantly higher than chance. The null hypothesis stated that the models would not perform better than random guessing (AUC ≤ 0.5), whereas the alternative hypothesis was that the models would achieve higher discrimination (AUC > 0.5). Based on these hypotheses, with a significance level of 0.05, and a power of 0.8, the estimated minimum sample size was approximately 63–65 patients, requiring at least 23 D-TGCT and 40 L-TGCT cases. Therefore, the current dataset of 84 patients (54 L-TGCT and 30 D-TGCT) provides adequate statistical power to evaluate model performance.

All statistical analyses were performed using SPSS version 26.0 (IBM Corp., Armonk, NY, USA). To assess differences in demographic and clinical data between the training and test cohorts, statistical comparisons were conducted using independent *t*-tests for continuous variables and Chi-square or Fisher’s exact tests for categorical variables. To assess interobserver agreement of tumor segmentation, it was quantified using the Dice similarity coefficient (DSC). Model performance was evaluated by calculating sensitivity, specificity, accuracy, and AUC. These metrics were computed for both the MLR and RF models in the training and test cohorts. A *p*-value < 0.05 was considered statistically significant.

## 3. Results

### 3.1. Patient Demographics

The study included a total of 84 patients who were divided into a training set (*n* = 52) and a test set (*n* = 32). The sex distribution was not significantly different between the two groups (*p* = 0.149), with males constituting 34.6% of the training set and 53.1% of the test set. Similarly, the mean age in the training set was 38.4 ± 16.3 years and in the test set it was 34.2 ± 14.2 years, showing no significant difference (*p* = 0.231). Regarding TGCT subtypes, the distribution of localized-type and diffuse-type tumors was also not significantly different between the two groups (*p* = 0.178). In the training set, 67.3% of tumors were localized-type, and in the test set, 50.0% were localized-type. These findings confirmed that the training and test sets were well-balanced with respect to these key patient characteristics. Patient demographics for the training and test cohorts are summarized in Table 1.

### 3.2. Radiomics Model Training and Performance

Before the radiomics analysis, we evaluated interobserver agreement of tumor segmentation. The median DSC between two readers was 0.73 (interquartile range, 0.55–0.79), indicating moderate to good agreement.

Two machine learning classifiers—MLR and RF—were developed to differentiate L-TGCT from D-TGCT based on T2WI radiomics features. Feature selection for the MLR model followed the *one-in-ten* rule to avoid overfitting, resulting in the identification of three discriminative T2WI–derived features. The first feature, wavelet-LHL_glszm_GrayLevelNonUniformity, measures the variability of gray-level intensities within the image, with higher values indicating tissue heterogeneity. Such heterogeneity may be associated with complex tumor microstructure, variable cellularity, or heterogeneous hemosiderin deposition. The second feature, wavelet-HLL_gldm_LowGrayLevelEmphasis, reflects the relative distribution of low-intensity voxels in the image. This feature might be relevant for identifying specific tumor types that appear with darker signal intensity on T2WI, which is potentially indicative of hemosiderin-rich regions or dense fibrous stroma. The third feature, square_firstorder_Median, represents the median pixel intensity after applying a square transformation, providing a measure of the central tendency that is robust to outliers and offers an overall measure of lesion brightness. It may correlate with tumor cellularity and water content.

Table 2 and Table 3 show the feature importance ranking for the MLR and RF models, respectively. As shown in Table 2 and Table 3, the feature importance rankings differed between the two models. While T2_wavelet-LHL_glszm_GrayLevelNonUniformity was ranked highly in both models, other features were significant predictors only in the RF model, likely due to its non-linear relationship with the outcome.

**Figure 3:**
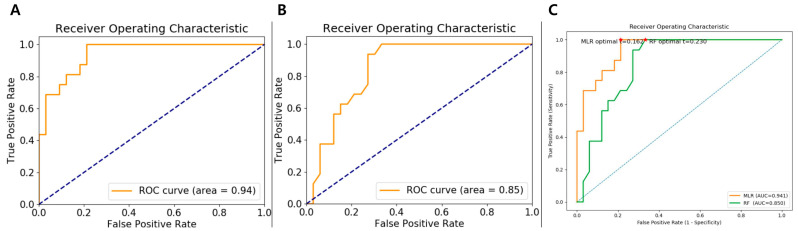
The AUC of MLR (**A**) and RF (**B**) model applied on training cohort for prediction of D-TGCT. (**C**) Comparison of ROC curves with decision thresholds (red stars). AUC, area under the curve; D-TGCT, diffuse-type tenosynovial giant cell tumor; MLR, multivariate logistic regression; RF, random forest; ROC, receiver operating characteristic.

In the training set, the MLR model demonstrated excellent classification performance, achieving an AUC of 0.94 (95% CI:0.65–1.00), sensitivity of 75.0% (95% CI: 0.50–0.89), specificity of 90.9% (95% CI: 0.74–0.96), and accuracy of 85.7% (95% CI: 0.73–0.92). The RF model, trained with 10-fold cross-validation, also achieved strong discrimination with a training set AUC of 0.85 (95% CI: 0.54–1.00), sensitivity of 43.8% (95% CI: 0.23–0.66), specificity of 87.9% (95% CI: 0.72–0.95), and accuracy of 73.5% (95% CI: 0.59–0.83). The performance metrics for both models are presented in Table 4. ROC curves for both the MLR and RF models and comparison of ROC curves with decision thresholds in the training cohort are presented in Figure 3.

All radiomics features from both T2WI and CE T1WI images were initially extracted and subjected to feature selection. However, only features derived from the T2WI images were retained in the final MLR model, while CE T1WI-derived radiomics features were excluded. This likely because T2WI features exhibited stronger associations with the subtype differentiation, whereas CE T1WI features did not provide additional independent predictive value. Consequently, the final MLR model is based solely on T2WI features.

### 3.3. Validation of the Radiomics Model

When applied to the test cohort, the performance of the two models diverged. The MLR model, despite its high training AUC, showed a marked decline in generalization, with the AUC dropping to 0.60 (95% CI: 0.23–0.97). This substantial decrease suggests overfitting, whereby the model captured patterns and noise specific to the training set that were not reproducible in unseen data. Its sensitivity, specificity, and accuracy in the test cohort were 62.5% (95% CI: 0.38–0.81), 60.6% (95% CI: 0.43–0.75), and 61.2% (95% CI: 0.47–0.73), respectively, further reflecting reduced performance. Given that an AUC of 0.60 is only slightly above random chance, the MLR model’s predictive value is limited and unlikely to be clinically useful in its current form.

**Figure 4:**
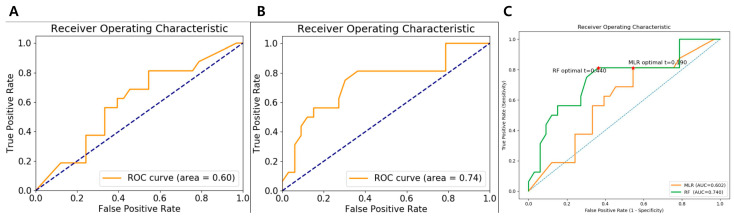
The AUC of MLR (**A**) and RF (**B**) model applied on test cohort for prediction of D-TGCT. (**C**) Comparison of ROC curves with decision thresholds (red stars). AUC, area under the curve; D-TGCT, diffuse-type tenosynovial giant cell tumor; MLR, multivariate logistic regression; RF, random forest; ROC, receiver operating characteristic.

In contrast, the RF classifier demonstrated more stable generalization. Its test-set AUC of 0.74 (95% CI: 0.39–1.00) represented a smaller decline from its training performance (AUC, 0.85) compared with the MLR model. The RF model also maintained favorable sensitivity, specificity, and accuracy values (56.2% [95% CI: 0.33–0.76], 72.7% [95% CI: 0.55–0.84], and 67.3% [95% CI: 0.53–0.78], respectively) on the test cohort, underscoring its relative robustness. This superior stability may be attributed to the inherent properties of ensemble learning—aggregating multiple decision trees tends to reduce variance and mitigate overfitting, enabling more consistent performance across datasets. The performance metrics for both models are presented in Table 5. ROC curves for both the MLR and RF models and comparison of ROC curves with decision thresholds in the test cohort are presented in Figure 4.

Overall, these findings indicate that although the MLR model achieved higher apparent accuracy in the training phase, the RF model provided better robustness and reliability on validation. Therefore, the RF classifier appears to be a more suitable candidate for clinical translation in differentiating L-TGCT from D-TGCT.

## 4. Discussion

In this study, we developed and validated two MRI-based radiomics models—MLR and RF—to predict D-TGCT. The observed performance discrepancy between the two models was notable. The MLR model achieved higher discrimination in the training phase but demonstrated a substantial performance decline on independent testing, suggesting overfitting. In contrast, the RF classifier, despite lower training accuracy, showed more stable performance in the test cohort, indicating better generalizability. These findings highlight the critical issue of model overfitting, particularly when dealing with complex, high-dimensional radiomics data [10,27]. These results underscore the potential utility of ensemble learning methods in radiomics-based MSK tumor classification.

D-TGCT has distinct pathological features compared to its localized counterpart, L-TGCT, which are often visible on MRI [7]. Previous studies have shown that D-TGCT often exhibits more infiltrative growth patterns, a larger extent, and heterogeneous signal intensity due to variable presence of hemosiderin, fibrous stroma, and cellularity, findings that may translate into measurable radiomic differences on MRI [4,5,7]. Our MLR model’s selected T2WI-derived features—GrayLevelNonUniformity, LowGrayLevelEmphasis, and Median intensity—directly correspond to these known pathological characteristics [7]. GrayLevelNonUniformity reflects the variability of voxel intensity, a metric for heterogeneity, while LowGrayLevelEmphasis and Median intensity relate to the overall signal distribution, which is influenced by factors such as hemosiderin deposition and fibrous stroma [20]. However, while logistic regression can provide interpretable feature associations [28], its linear assumptions likely prevented it from capturing complex, nonlinear interactions. This limitation is a known challenge for traditional statistical models in radiomics, where the relationships between texture features and clinical outcomes are often intricate and non-linear [29,30]. Such inability to model complex feature interactions is a major reason for MLR’s substantial performance decline on the independent test set, a classic sign of overfitting [10]. In addition, the MLR models’ AUC of 0.60 on the test cohort indicates that its predictive performance was only marginally better than chance, underscoring its limited clinical applicability and highlighting the need for more advanced modeling approaches.

The RF model’s robust performance can be attributed to its inherent design, which is well-suited for radiomics analysis. By building multiple uncorrelated decision trees and aggregating their predictions, RF effectively mitigates the risk of overfitting and reduces model variance [24,29,31]. This is particularly advantageous in studies with a relatively small sample size, which is common for rare tumors like D-TGCT [32]. RF’s ability to handle high-dimensional, correlated features without requiring strict linearity assumptions makes it a powerful tool for analyzing complex texture data extracted from medical images [33]. The smaller drop in AUC between our training and testing cohorts for the RF model is a strong indicator of its potential for clinical implementation, where unseen data variability is inevitable and generalizability is paramount [34].

To the best of our knowledge, this is the first study to apply radiomics to predict D-TGCT using conventional MRI sequences. This study makes several contributions to the existing literature on TGCT and MSK tumors. First, we propose a novel quantitative approach to a diagnostic challenge for TGCT evaluation using conventional MRI that may support pretreatment subtype differentiation. Furthermore, by comparing regression-based and machine learning classifiers, we highlight the importance of selecting appropriate machine learning models for radiomics analysis. The superior and more stable performance of the RF model underscores the value of ensemble learning methods in handling the complexities of high-dimensional radiomics data with limited sample sizes, providing a crucial lesion for future studies in MSK oncology, especially rare tumors.

Despite these promising findings, several limitations should be acknowledged. First, this was a single-center retrospective study, and the relatively small cohort size, although typical for rare tumors, may have limited the statistical power and increase the risk of type II errors. In particular, the relatively small number of patients (*n* = 84) inevitably increases the risk of overfitting, which was observed in the MLR model of our study. This limitation arises partly from the Syngo.Via Radiomics Prototype, which does not implement cross-validation for logistic regression. In contrast, the RF classifier includes embedded cross-validation, likely contributing to its more stable performance. Second, a notable limitation of our study is the relatively low sensitivity of both the MLR and RF models in the test cohort, indicating that some D-TGCT cases could be misdiagnosed as L-TGCT. This may lead to suboptimal surgical planning or higher recurrence risk. The lower sensitivity may be attributed to the limited sample size, class imbalance, or reliance solely on T2WI features. We will consider incorporating multimodal data, including clinical variables and additional multiparametric MRI sequences, such as DWI or DCE-MRI, to build a more comprehensive model with increased predictive power [35,36]. Third, due to the class imbalance in our dataset (53 vs. 30 cases), accuracy alone may be insufficient; while AUC was reported, more detailed class-specific metrics were not directly available from the prototype. Fourth, variation in MRI scanners and acquisition protocols, though partially mitigated by image preprocessing, may still have influenced radiomics feature stability [37,38]. Fifth, our study only conducted internal validation using a single institution’s data. The absence of external validation limits the ability to confirm the generalizability of our findings across different institutions and imaging protocols. Future multicenter studies including external validation cohorts are essential to establish the robustness and clinical utility of these radiomics models.

## 5. Conclusions

Our study suggests that MRI-based radiomics has the potential to predict D-TGCT, with the RF classifier showing relatively greater robustness and generalizability compared to MLR. These results indicate that advanced machine learning techniques, particularly ensemble methods, could be beneficial in musculoskeletal radiomics for addressing challenges of small sample sizes and complex feature relationships. Our findings will inform future efforts toward integrating these methods into preoperative planning and individualized patient management. Further rigorous, prospective, and multi-institutional validation is imperative before these radiomics models can be adopted into routine clinical practice.

## Figures and Tables

**Table 1:** Demographic findings in the training and test sets.

Characteristics	Training Set	Test Set	*p*-Value
	(*n* = 52)	(*n* = 32)	
Sex			0.149
Male	18 (34.6%)	17 (53.1%)	
Female	34 (65.4%)	15 (46.9%)	
Age (years)	38.4 ± 16.3	34.2 ± 14.2	0.231
TGCT subtypes			0.178
Localized-type	35 (67.3%)	16 (50.0%)	
Diffuse-type	17 (32.7%)	16 (50.0%)	

TGCT, tenosynovial giant cell tumor.

**Table 2:** Top 3 most important features from the MLR model.

Rank	Feature	OR (Odds Ratio)	95% CI (Lower–Upper)	*p*-Value
1	T2_wavelet-LHL_glszm_GrayLevelNonUniformity	5.291	1.381–20.267	0.015
2	T2_wavelet-HLL_gldm_LowGrayLevelEmphasis	5.077	1.506–17.114	0.0088
3	T2_square_firstorder_Median	3.599	0.858–15.101	0.080

**Table 3:** Top 10 most important features from the RF model.

Rank	Feature	Importance (All)	Mean (Folds)	Std (Folds)
1	T2_wavelet-LHL_glszm_GrayLevelNonUniformity	0.024	0.014	0.008
2	CE_wavelet-HHL_glszm_SmallAreaHighGrayLevelEmphasis	0.020	0.005	0.005
3	T2_wavelet-LHH_firstorder_90Percentile	0.017	0.005	0.005
4	T2_original_firstorder_10Percentile	0.016	0.016	0.008
5	T2_wavelet-LLL_firstorder_Mean	0.015	0.003	0.003
6	T2_wavelet-HLL_glcm_ClusterShade	0.011	0.007	0.004
7	T2_logarithm_glszm_GrayLevelNonUniformity	0.011	0.003	0.003
8	T2_original_firstorder_TotalEnergy	0.010	0.003	0.003
9	CE_wavelet-LHL_glszm_SizeZoneNonUniformity	0.009	0.005	0.004
10	T2_wavelet-HLL_firstorder_Entropy	0.009	0.015	0.007

**Table 4:** Performance of MLR and RF models in the training cohort.

Model	AUC (95% CI)	Sensitivity (95% CI)	Specificity (95% CI)	Accuracy (95% CI)
MLR	0.94 (0.65–1.00)	75.0% (0.50–0.89)	90.9% (0.74–0.96)	85.7% (0.73–0.92)
RF	0.85 (0.54–1.00)	43.8% (0.23–0.66)	87.9% (0.72–0.95)	73.5% (0.59–0.83)

**Table 5:** Performance of MLR and RF models in the test cohort.

Model	AUC (95% CI)	Sensitivity (95% CI)	Specificity (95% CI)	Accuracy (95% CI)
MLR	0.60 (0.23–0.97)	62.5% (0.38–0.81)	60.6% (0.43–0.75)	61.2% (0.47–0.73)
RF	0.74 (0.39–1.00)	56.2% (0.33–0.76)	72.7% (0.55–0.84)	67.3% (0.53–0.78)

## Data Availability

The dataset is available on request from the authors.
